# Qualities of Waters in St. George's, Hanover Square
*Report on the Quality of the Waters used in the In-Ward of St. George's, Hanover Square. By R. Druitt, M.D. 1856.


**Published:** 1857-04

**Authors:** 


### QUALITIES OF WATERS IN ST. GEORGE'S, HANOVER SQUARE.*

Dr. Druitt represents that the best test of the quality of the organic matter dissolved in water is its power of keeping sweet if exposed to air and light in a warm place. He thinks that the existence in water of living animals and vegetables is an unfair test of the purity of the water originally, inasmuch as pure water exposed to air, light, and sunshine, would soon teem with animals and plants. In the purity of the water of the Grand Junction Company, Dr. Druitt has "the utmost confidence."

Dr. Aldis, the colleague of Dr. Druitt in his sanitary labours, has given a report on the sanitary condition of his district, to which we shall again refer. Dr. Hassall has published a new work on *The Adulteration of Foods and Medicines*, not to supersede his previous work, but to place before the people the easiest modes of detecting adulterations. This work adds to the already well earned reputation of the writer. Dr. Huxley, medical superintendent of the Kent Lunatic Asylum, has issued his annual report, from which it appears that Kent has now "900 acknowledged lunatics to provide for." Dr. Dickson, superintendent of the Manchester Royal Lunatic Asylum, favours us with a similar document. His results show the recoveries to be nearly 68 per cent. in 160 admitted. His treatment consists in nourishing the strength of the patients, instead of lowering it, and in maintaining a strict Hygienic system. Dr. W. F. Daniell has a most interesting paper on the *Copals of Western Africa*. Dr. Richardson has brought out, in an enlarged form, and with an additional chapter on treatment, his essay on the *Hygienic Treatment of Pulmonary Consumption*.

---

* Report on the Quality of the Waters used in the In-Ward of St. George's, Hanover Square. By R. Druitt, M.D. 1856.

